# Importance of metabolic and immune profile as a prognostic indicator in patients with diabetic clear cell renal cell carcinoma

**DOI:** 10.3389/fonc.2023.1280618

**Published:** 2023-10-20

**Authors:** Xiangyu Cheng, Yanlian Hou

**Affiliations:** ^1^Department of Urology, The Second Affiliated Hospital of Shandong First Medical University, Taian, Shandong, China; ^2^Department of Endocrinology, The Second Affiliated Hospital of Shandong First Medical University, Taian, Shandong, China

**Keywords:** metabolism, diabetes, clear cell renal cell carcinoma, immune profile, gene set enrichment analysis

## Abstract

**Background:**

ccRCC, also known as clear cell renal cell carcinoma, is a cancer that is highly metabolically active and has a strong connection with the immune system. The objective of this research was to investigate the correlation between pathways associated with metabolism, diabetes, immune infiltration, and their impact on the prognosis of ccRCC.

**Method:**

We conducted an extensive examination utilizing ssGSEA, ESTIMATE algorithm, WGCNA, and GSVA for gene set enrichment analysis, gene co-expression network analysis, and gene set variation analysis. An established prognostic model, utilizing immune-related WGCNA findings, was evaluated for its association with clinical characteristics and the tumor microenvironment (TME).

**Result:**

The ssGSEA effectively categorized ccRCC into groups based on low and high metabolism. Strong associations were observed between scores related to metabolism and immune scores, ESTIMATE scores, stromal scores, and gene expression related to HLA. The analysis conducted by WGCNA revealed a module called the ‘yellow module’ that exhibited a significant correlation with the infiltration of immune cells and the survival rates of patients. A risk model was developed, demonstrating reliable predictive performance for patient survival outcomes. The risk model also correlated significantly with immune scores and HLA-related gene expressions, suggesting potential immune evasion mechanisms. The analysis of mutations in TCGA data revealed the mutational patterns of ccRCC, and there was a significant correlation between the risk score and clinical characteristics. The GSVA analysis revealed a notable enrichment of pathways associated with cancer in patients at high risk. Finally, in order to evaluate the role of CX3CL1 in renal cell carcinoma cells, we then performed the cell proliferation assays. The results demonstrated that the over expression of CXCL1 could promote the cell proliferation ability in renal cell carcinoma cells.

**Conclusion:**

Our findings provide a novel perspective on the interactions between diabetes, metabolic pathways, and the immune landscape in ccRCC. The predictive value of the prognostic model established in this research has the potential to guide the development of new therapeutic and prognostic approaches for patients with ccRCC.

## Introduction

Diabetes is a long-term condition that arises when the pancreas fails to generate sufficient insulin or when the body is unable to properly utilize the insulin it generates (1). The hormone insulin plays a crucial role in controlling the levels of sugar in the bloodstream. Insufficient insulin can result in elevated blood glucose levels, which can contribute to numerous complications such as kidney disease, heart disease, stroke, as well as potential harm to the eyes and nerves (2). Diabetes can be classified into two primary forms: Type 1 Diabetes and Type 2 Diabetes. Typically, Type 1 diabetes is identified in kids and individuals in their early adulthood, and it is distinguished by the body’s incapacity to generate insulin (3). This type requires regular insulin injections to regulate blood sugar levels. Insulin resistance is a defining feature of Type 2 Diabetes, which is the prevailing variant primarily impacting adults. In this scenario, the body continues to generate insulin but fails to utilize it effectively (4). As time passes, the pancreas might cease the production of sufficient insulin, leading to an increase in blood sugar levels. Diabetes management involves monitoring and managing blood glucose levels, adopting a healthy lifestyle with regular physical activity, and a balanced, nutritious diet (5). Medication or insulin therapy may be necessary depending on the type of diabetes and its progression. It is crucial to prevent or control diabetes because it has a significant effect on general well-being and quality of life, and it is linked to various complications and co-existing medical conditions (6). Therefore, it remains a crucial field of continuous investigation and public health initiatives.

Globally, diabetes and renal cell carcinoma (RCC), which is another term for kidney cancer, are two major concerns impacting public health. Recent studies have indicated a potential link between these two disorders (7). Diabetes is a long-term condition marked by the body’s incapability to adequately generate or utilize insulin, leading to increased glucose levels in the bloodstream (8). Over a period of time, inadequately managed diabetes can result in different complications, such as cardiovascular disease, cerebral vascular accident, neuropathy, and renal disease (9). In contrast, kidney cancer is a form of cancer that originates in the kidneys. Although the precise reason behind kidney cancer remains uncertain, specific factors that increase the risk include tobacco use, being overweight, and having elevated blood pressure (10). According to recent epidemiological research, individuals with diabetes have been found to have a higher likelihood of developing kidney cancer. This indicates that diabetes could potentially be a separate contributing factor to the occurrence of this particular cancer (11). The possibility of this connection could be attributed to the chronic harm caused by elevated blood sugar levels, potentially leading to alterations at the cellular level that enhance the likelihood of developing cancer (12). Insulin resistance, a common occurrence in individuals with type 2 diabetes, can serve as an additional linking element since it results in elevated levels of insulin and insulin-like growth factor present in the bloodstream, potentially fostering the development of tumors (13). Nevertheless, additional investigation is required to further examine and comprehend the mechanisms that underlie the correlation between diabetes and renal cancer. Such understanding can provide critical insights into prevention strategies and therapeutic interventions for both conditions.

This article will employ diverse bioinformatics methods to examine the molecular-level connection between diabetes and kidney cancer. The process involves extracting information from openly accessible databases, examining variations in gene expression, studying networks of protein interactions, and conducting an analysis of functional enrichment. The goal is to discover possible biomarkers, disrupted pathways, and treatment targets that could clarify the observed link between diabetes and kidney cancer. The knowledge obtained from this bioinformatics examination will not just enhance our comprehension of the intricate interaction between these two illnesses but may also aid in the creation of preventive approaches and innovative therapies.

## Method

### Gathering information from the cancer genome atlas database

The study obtained data from The Cancer Genome Atlas (TCGA), a comprehensive repository maintained by the National Cancer Institute and the National Human Genome Research Institute. TCGA offers genomic and clinical information for more than 30 cancer types. We acquired data from the TCGA Kidney Renal Clear Cell Carcinoma (KIRC) project for the precise examination of renal clear cell carcinoma (ccRCC). Our study included all participants in the KIRC project who possessed both genomic sequencing data and corresponding clinical information.

### Gene set enrichment analysis performed on a single sample using ssGSEA method

The enrichment scores for each gene set in each sample were obtained using ssGSEA, which measures the extent to which the genes in a specific gene set are collectively upregulated or downregulated in a sample. To conduct our investigation, we utilized the GSVA tool in the R programming language to carry out ssGSEA. The ssGSEA scores indicate the extent to which genes within a specific set are collectively upregulated or downregulated in a given sample. Hence, the ssGSEA score measures the enrichment of a set of genes in an individual sample, enabling us to compare the activation of the gene set among various samples.

### Analysis of gene ontology

To conduct GO analysis of the identified DEGs, we utilized the Database for Annotation, Visualization, and Integrated Discovery (DAVID, v6.8). The analysis of gene ontology (GO) includes three distinct categories: Biological Process (BP), Cellular Component (CC), and Molecular Function (MF). The objective of the GO analysis is to offer a collection of top-level functional annotations in order to uncover the characteristics of genes and gene products.

### Analysis of pathways using the Kyoto encyclopedia of genes and genomes

To further explore the biological pathways associated with the differentially expressed genes (DEGs), we conducted KEGG pathway analysis utilizing the KEGG Orthology Based Annotation System (KOBAS, v3.0). KEGG pathway analysis can provide insight into the larger biological systems in which these genes operate and their potential roles in disease.

### Immune-related weighted gene co-expression network analysis

In order to investigate the co-expression patterns of immune-related genes in our dataset, we utilized Weighted Gene Co-Expression Network Analysis (WGCNA). To begin, immune-related genes were identified. From the ImmPort database, we acquired an extensive compilation of genes associated with the human immune system. This list was cross-referenced with our dataset to extract the expression data for these genes. Next, we built a gene co-expression network utilizing the ‘WGCNA’ software in R (version 4.1.0). Initially, a matrix was calculated that displayed the pairwise correlations among every combination of genes. The adjacency matrix was subsequently converted into a topological overlap matrix (TOM), which quantifies the connectivity of a gene in the network by summing its adjacency with all other genes. In order to group genes with comparable expression patterns into modules, average linkage hierarchical clustering was performed using the TOM-based dissimilarity measure, with a minimum size of 30 for the resulting gene groups.

### Quantification of immune cell infiltration using multiple algorithms

In order to offer a complete perspective on the infiltration of immune cells in our dataset, we utilized multiple computational algorithms, including CIBERSORT, TIMER, quanTIseq, and xCell. Using gene expression data, these tools estimate the prevalence of immune cell populations. The estimation of the proportion of 22 varieties of infiltrating immune cells was conducted using CIBERSORT, an algorithm for deconvolution. The LM22 gene signature was utilized with 1000 permutations. Samples that had a CIBERSORT output with a p-value less than 0.05 were selected for additional analysis. The TIMER online tool was utilized to estimate the prevalence of six different types of immune cells (B lymphocytes, CD4+ T lymphocytes, CD8+ T lymphocytes, neutrophils, macrophages, and dendritic cells). The data on gene expression was uploaded to the TIMER web portal for analysis. The fractions of ten immune cell types were estimated using quanTIseq. The raw gene expression data was directly used to run this tool without the need for a pre-processing step. The relative proportions of 64 immune and stromal cell types were deduced using xCell. It utilizes gene signatures and a spillover compensation technique to generate cell scores, which were converted into cell proportions. All algorithms were run using default parameters unless otherwise specified. Correlations were evaluated by comparing the results from each method and examining the inferred proportions of immune cell types.

### Analysis of differential expression

The differential expression analysis between tumor and normal samples was conducted using the edgeR package in R. Initially, the count data was normalized by employing the TMM (Trimmed Mean of M values) method. After normalizing the data, the differential expression analysis was conducted using the exactTest function in edgeR.

### Feature selection and prognostic model construction

The training set was used to assess the correlation between gene expression and overall survival using Cox regression analysis. Genes that had a p-value less than 0.05 were deemed to be statistically significant. Afterwards, a Cox regression analysis with Lasso penalty was conducted to identify the most valuable gene characteristics for prognosis and prevent overfitting. The Lasso Cox regression model was used to derive the regression coefficient for each gene’s expression level, which was then used to establish a risk score formula for predicting overall survival.

### Verifying the predictive model

Based on the risk score median, patients were categorized into groups of high-risk and low-risk. To compare the difference in survival between the high-risk and low-risk groups, we performed Kaplan-Meier survival analysis and log-rank tests. To evaluate the forecast model’s predictive precision, a time-sensitive analysis was conducted using a receiver operating characteristic (ROC) curve. Using the same approach, the testing set was used to further validate the prognostic model.

### Development of a nomogram through multivariate Cox regression analysis

Multivariate Cox regression analysis was conducted to assess the autonomy of the prognostic model from additional clinical characteristics. A clinical tool was developed by combining the risk score and clinical factors for practical application.

### Analysis of mutation data from TCGA

We obtained somatic mutation data for clear cell renal cell carcinoma (ccRCC) samples from The Cancer Genome Atlas (TCGA) database by utilizing the TCGAbiolinks package in R. This package simplifies the process of retrieving data, guaranteeing the utilization of current and extensive information sourced from TCGA. The original mutation data, which was provided in MAF (Mutation Annotation Format) files, underwent processing using the R package called ‘maftools’. Maftools is a specialized tool created for thorough and integrated analysis of mutation data. In order to obtain valuable information from this data, we initially conducted an annotation process, in which every mutation was classified based on its genomic position and impact on the related protein product. Afterwards, we computed the mutation rate for every gene, which refers to the count of samples where each gene experienced a mutation, along with the specific type of mutation that took place. Genes with the highest mutation frequencies were identified and ranked. Finally, to visualize the mutational landscape of ccRCC, we generated waterfall plots, which provide an overview of the mutation distribution across samples and the frequency of different mutation types. This helps in understanding the genomic instability and identifying potential driver mutations in ccRCC.

### Analysis of gene set variation

To determine alterations in pathway activity among the samples, the GSVA package in R software was utilized, employing GSVA, an unsupervised and non-parametric method. By converting a matrix of genes by samples into a matrix of gene sets by samples, GSVA estimates the variability of gene set enrichment across the samples in the expression dataset. GSVA utilized the gene sets from the Molecular Signatures Database (MSigDB). GSVA scores were calculated and compared across the different groups, with significantly different scores indicating differential pathway activities.

### Cell culture

The American Type Culture Collection (ATCC) provided the human renal cancer cell lines (786-O and Caki-1) through purchase. Proper growth and proliferation of cells were ensured by maintaining them in suitable culture conditions. The cells were grown in RPMI 1640 solution (Gibco, USA), which was enriched with 10% fetal bovine serum (FBS, Gibco, USA), 100 U/mL penicillin, and 100 µg/mL streptomycin (Gibco, USA). This was done in a 37°C environment with 5% CO2, maintaining a humid atmosphere.

### Colony formation assay

A defined number of cells were seeded into each well of a 6-well plate. Plates were gently rocked to ensure even distribution of cells and then incubated at 37°C in a humidified incubator with 5% CO₂. Cells were allowed to grow for a specific duration, typically ranging from 7 to 14 days, or until visible colonies could be observed. During this period, the medium was not changed to avoid disturbing the developing colonies. After incubation, the medium was removed, and the cells were gently washed with phosphate-buffered saline (PBS). Colonies were fixed with methanol or 4% paraformaldehyde for 15 minutes at room temperature. After fixation, cells were stained with 0.5% crystal violet solution in 20% methanol for 30 minutes.

### Statistical analysis

R software (version 4.0.2) was used for all statistical analyses. A p-value less than 0.05, indicating statistical significance, was deemed significant in both directions.

## Result

### The ssGSEA examination unveiled the pathways associated with metabolism in the cohort of clear cell renal cell carcinoma

**Figure 1:**
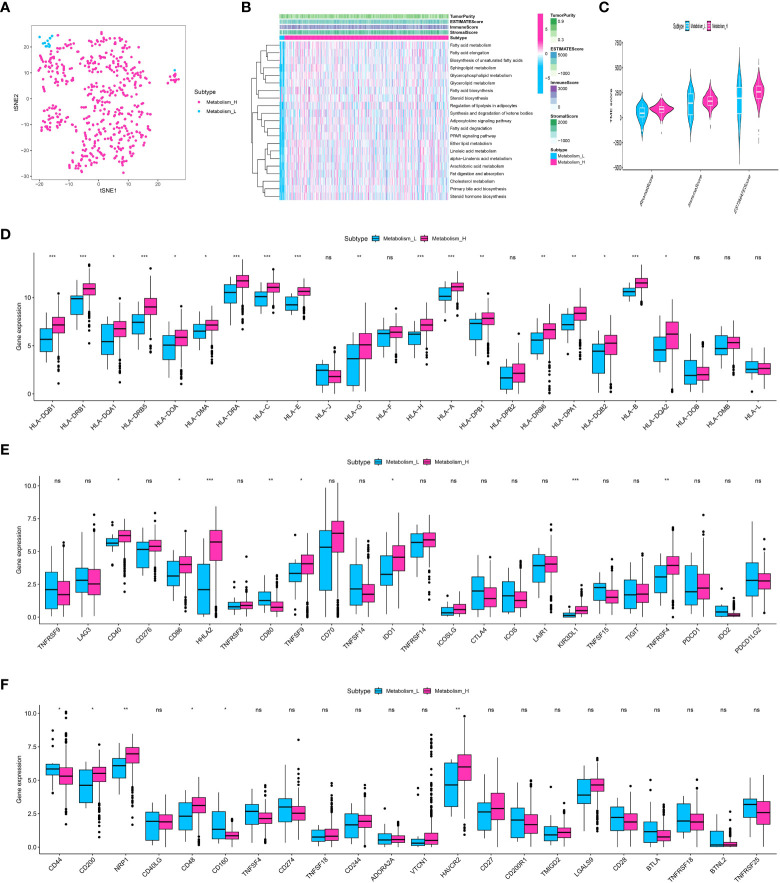
**(A)** The ccRCC cohort was divided into low and high-metabolism groups based on the ssGSEA analysis; **(B)** The heatmap demonstrated the enriched metabolism-related score in ccRCC patients; **(C)** The correlation analysis revealed the correlation between TME and metabolism-related scores; **(D)** The correlation analysis revealed the correlation between HLA-related genes and metabolism-related scores; **(E, F)** The correlation analysis revealed the correlation between immune checkpoint-related genes and metabolism-related scores. * p<0.05, ** p<0.01, *** p<0.001. ns, not significant.

**Figure 2:**
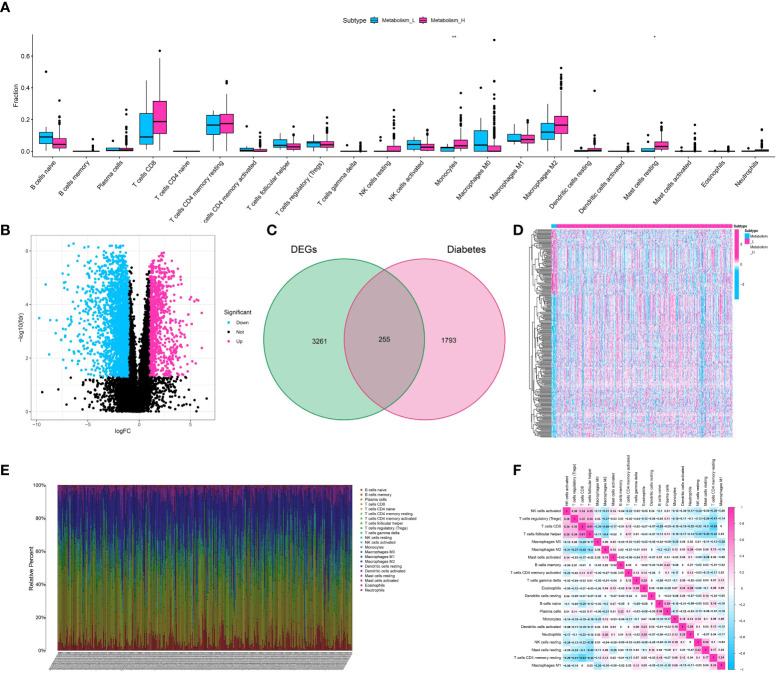
**(A)** The correlation analysis showed the correlation between immune-related cells and metabolism-related scores; **(B)** The volcano diagram showed the differentially expressed genes between low- and high-metabolism groups; **(C)** The venn diagram showed the diabetes-related genes involved in differentially expressed genes; **(D)** The heatmap demonstrated the expression level of differentially expressed genes between metabolism-low and metabolism-high groups; **(E)** The immune infiltration analysis showed the distribution of multiple immune-related genes in renal cell carcinoma cohort; **(F)** The correlation analysis showed the correlative scores between different immune-related cells.

ssGSEA was used to measure the enrichment levels in the study of lipid-related metabolic pathways. Numerous routes were analyzed, covering procedures like synthesis of fatty acids, elongation, breakdown, production of steroids and bile acids, metabolism of glycerophospholipids, metabolism of arachidonic acid, metabolism of linoleic acid, metabolism of alpha-linolenic acid, metabolism of sphingolipids, signaling of adipocytokines, regulation of adipocyte lipolysis, digestion and absorption of fats, metabolism of cholesterol, synthesis of unsaturated fatty acids, metabolism of glycerolipids, and the pathway of PPAR signaling. Following this, utilizing the overall score related to metabolism, the clear cell renal cell carcinoma was effectively categorized into groups with low and high metabolism (**Figure 1A**). Furthermore, the heatmap showcased the heightened degree of metabolic pathways enrichment in the cohort of clear cell renal cell carcinoma (**Figure 1B**). Additionally, we assess the association between the metabolic score and the tumor microenvironment (TME), encompassing the immune score, ESTIMAT score, and stromal score (**Figure 1C**). Additionally, the analysis also involved the inclusion of genes related to human leukocyte antigen (HLA) for correlation purposes. The correlation analysis showed that the expression level of certain HLA-associated genes, including HLA-DQB1, HLA-DRB1, HLA-DRB5, HLA-DRA, HLA-C, HLA-E, and others, was identified (**Figure 1D**). Additionally, we assess the association between genes related to immune checkpoints and the score related to metabolism. The findings indicated that a score associated with metabolism could potentially provide guidance for immune checkpoint therapy, including but not limited to CD40, CD86, HHLA2, CD80, TNFSF9, IDO1, KIR3DL1, TNFRSF4, and others (**Figures 1E, F**). In addition, we assess the association between immune-related cells and scores related to metabolism. The findings indicated that a greater metabolic score enrichment is associated with an increased enrichment of monocytes and resting (**Figure 2A**).

### Discovering the genes associated with diabetes and the genes linked to metabolism in the cohort of clear cell renal cell carcinoma

To identify the genes related to diabetes and metabolism in the cohort of clear cell renal cell carcinoma, we initially conducted differential expression analysis using the ssGSEA analysis method. The patients with clear cell renal cell carcinoma were divided into low- and high-metabolism groups on average. The analysis of differential expression showed that a grand total of 3516 genes were identified as differentially expressed, comprising 1008 genes that were up-regulated and 2508 genes that were down-regulated (**Figure 2B**). To identify the genes linked to metabolism and diabetes, the venn diagram was utilized. The findings indicated that there is a strong correlation between diabetes and metabolism, with a total of 255 genes being closely associated (**Figure 2C**). Additionally, the heatmap illustrated the level of gene expression in the cohort of clear cell renal cell carcinoma, highlighting key genes (**Figure 2D**).

### The analysis of immune cell infiltration disclosed the extent of immune cell infiltration in the cohort of renal cell carcinoma

We utilized the ESTIMATE algorithm to measure the level of immune cell infiltration in ccRCC (clear cell renal cell carcinoma). The method produces three scores: the Stromal Score, the Immune Score, and the ESTIMATE Score. These scores indicate the presence of stromal cells, immune cells, and the overall tumor purity, respectively. The heatmap displayed the degree of immune cell infiltration for 22 different types of immune-related cells in the ccRCC cohort (**Figure 2E**). Furthermore, the correlation analysis additionally unveiled the immune (**Figure 2F**).

### Multiple immune-related cells were found to contain diabetes-related genes and genes associated with metabolism, as revealed by the WGCNA

**Figure 3:**
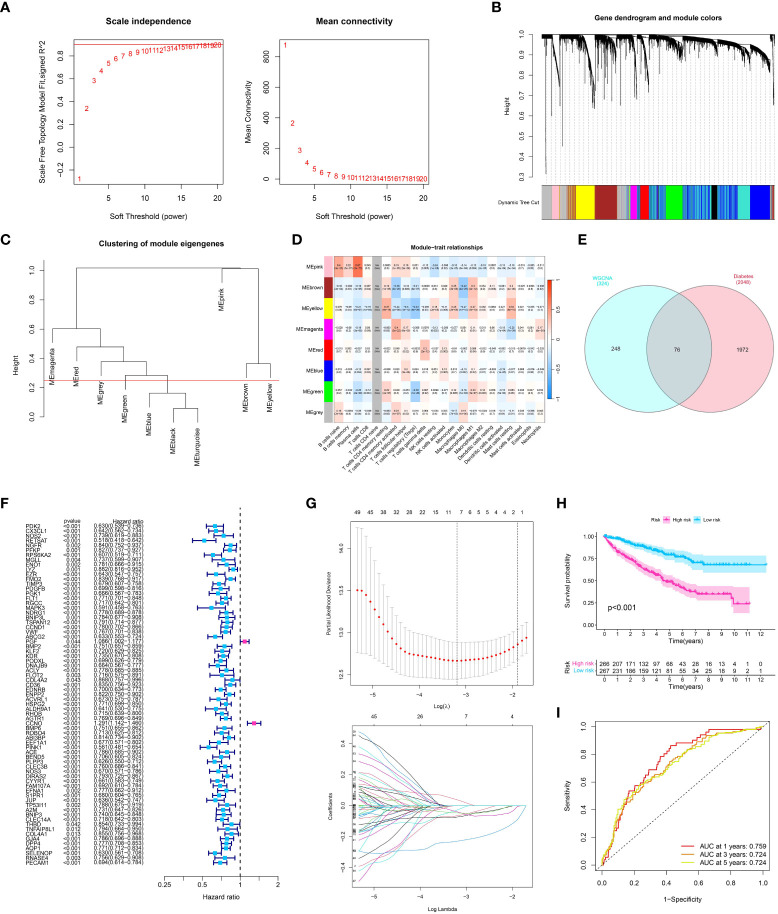
**(A)** Analysis for soft-thresholding selection, showing the scale-free fit index and mean connectivity for various soft-thresholding powers. The red line indicates the chosen soft-thresholding value; **(B, C)** Dendrogram of gene modules detected by WGCNA, with each color representing a unique gene module; **(D)** Module-trait relationship heatmap. Each cell displays the association and significance between the corresponding module and immune-related traits; **(E)** The venn diagram showed the co-expression genes in diabetes and module genes in WGCNA analysis; **(F)** The Univariate COX regression analysis showed the prognosis-related genes in ccRCC cohort; **(G)** The LASSO regression analysis; **(H)** The survival analysis showed the OS between low- and high-risk groups; **(I)** The time-dependent ROC curve showed the predictive value in one year, three year and five year.

Through the application of Weighted Gene Co-expression Network Analysis (WGCNA), an exploration was conducted to examine the correlation between gene modules and the immune composition of ccRCC. By building a network of gene co-expression, we discovered multiple clusters linked to the infiltration of immune cells in ccRCC. The ‘yellow module’, which was one of these modules, showed a noteworthy association with levels of immune cell infiltration as determined by ESTIMATE scores (**Figures 3A–D**). An extensive examination of this yellow module, which consists of 324 genes, showed an abundance of various immune-related cells, such as plasma cells, CD8 T cells, memory CD4 T cells, and others. This indicates a potential involvement in regulating the immune response within the ccRCC tumor microenvironment.

### Development of a prognostic model utilizing immune-associated WGCNA findings in ccRCC

After conducting the WGCNA analysis related to the immune system, we proceeded with a survival analysis to investigate the predictive significance of the identified gene module (**Figure 3E**). Patient overall survival showed significant associations with several candidate genes from the ‘yellow module’ using univariate Cox regression analysis and LASSO regression analysis (**Figures 3F, G**). Next, we applied a multivariate Cox regression analysis to these selected genes. Our prognostic model is based on the emergence of four genes as separate prognostic markers for ccRCC (all p < 0.05). The risk score was computed by considering the expression level of these genes, which were weighted by their corresponding regression coefficients obtained from the multivariate Cox regression model. Based on the risk score median, patients were categorized into groups of high-risk and low-risk. According to the log-rank test (p < 0.001), the high-risk group exhibited notably inferior overall survival compared to the low-risk group, as indicated by the Kaplan-Meier survival analysis (**Figure 3H**). The analysis of the receiver operating characteristic (ROC) curve showed that our risk score had a strong predictive accuracy for overall survival at 1-year, 3-year, and 5-year intervals (**Figure 3I**).

### The established prognostic model is used to create a prognostic nomogram, analyze risk curve, perform univariate and multivariate analyses, and generate ROC curves

**Figure 4:**
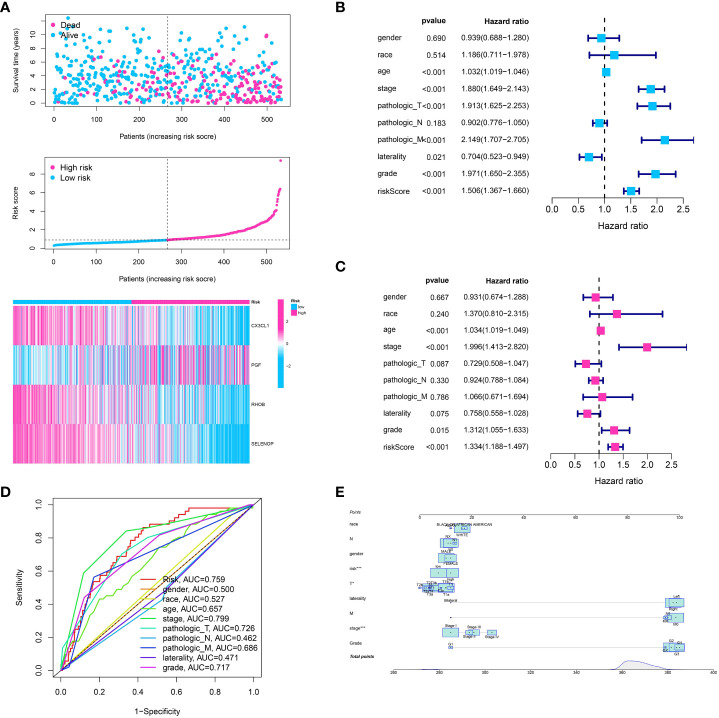
**(A)** The risk plot showed the ccRCC patients involved in low- and high-risk groups for ccRCC cohort; **(B)** The univariate independent prognostic analysis based on clinical features and risk models; **(C)** The multivariable independent prognostic analysis based on clinical features and risk models; **(D)** The ROC curve showed the predictive value for clinical features and risk model; **(E)** The nomogram showed the predictive value for risk model combined with clinical features.

After creating the predictive model, we constructed risk graphs to visually represent the risk profiles of the patients. The patients were arranged on the x-axis based on their increasing risk scores. The group at a higher risk, identified by scores indicating risk above the middle value, showed a significant rise in the occurrence of mortality events, indicating the potential usefulness of our model for predicting patient outcomes ***(*** **Figure 4A**). The prognostic model generated a risk score that showed a significant association with overall survival in univariate analysis (hazard ratio (HR) > 1, p < 0.001) (**Figure 4B**). This suggests that a higher risk score is linked to poorer survival outcomes. Additionally, survival was significantly correlated with other clinical characteristics including age, tumor stage, and tumor grade (all p < 0.05). In the analysis of multivariate Cox regression, the significance of our model’s risk score remained (HR > 1, p < 0.001), indicating that the prognostic importance of our model is unaffected by these additional clinical characteristics (**Figure 4C**). Hence, the hazard score serves as a separate predictive element for the overall lifespan of individuals with ccRCC. Next, we created receiver operating characteristic (ROC) curves to evaluate the accuracy of the model’s predictions. Our risk model demonstrated good predictive performance with an area under the curve (AUC) of 0.759 (**Figure 4D**). To achieve a prognostic model with enhanced predictive value, the construction of a nomogram was ultimately undertaken (**Figure 4E**).

### Immune infiltration analysis, risk model and tumor microenvironment correlation

**Figure 5:**
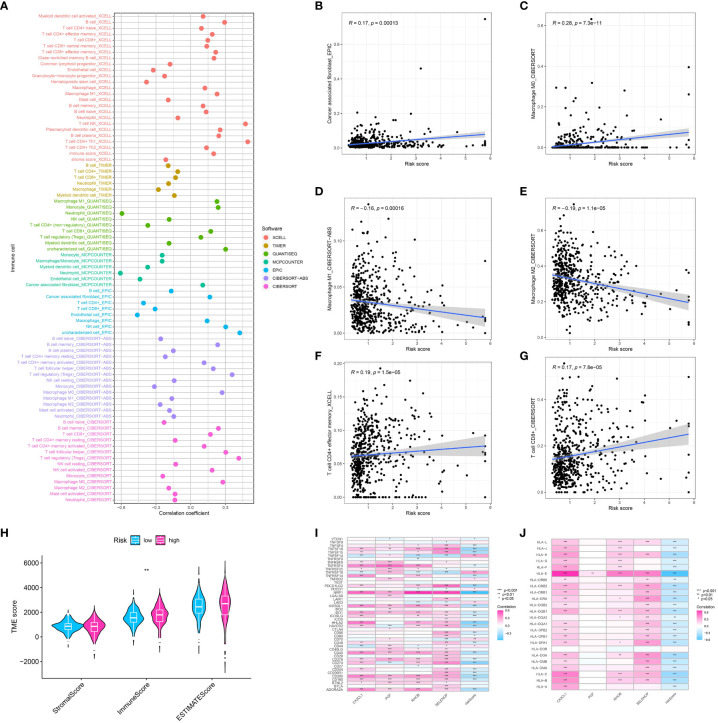
**(A)** The correlation analysis between immune cell infiltration and risk score using the multiple algorithm; The correlation between risk score and cancer -associated fibroblasts **(B)**, M0 macrophages **(C)**, M1 macrophage **(D)**, M2 macrophage **(E)**, memory CD4+ T cell **(F)**, CD8 + T cell **(G)**; **(H)** The correlation analysis between TME and risk score; **(I)** The correlation between genes involved in risk model and immune checkpoint-related genes; **(J)** The correlation between genes involved in risk model and HLA-related genes.

We applied several algorithms to perform immune infiltration analysis, including CIBERSORT, TIMER, QUANTISEQ, XCELL, and MCP-counter. Every algorithm showcased a distinct perspective of the immune terrain, offering a holistic comprehension of immune cell infiltration in ccRCC. The risk model identified substantial variances in the proportions of immune cells between the high and low-risk groups, as indicated by our analysis (**Figures 5A–G**). The risk model had a strong correlation with the TME, which underwent evaluation through the utilization of the ESTIMATE algorithm (**Figure 5H**). Significantly, there was a notable difference in immune scores between the groups classified as high-risk and low-risk. An elevated immune cell infiltration was indicated by the higher immune scores observed in the high-risk group. After examining the association between the risk model and genes related to immune checkpoints, it was discovered that the risk score showed a significant correlation with the expression of several checkpoint genes (**Figure 5I**). This may indicate a potential way for the immune system to evade detection in the group at high risk and propose potential targets for treatment. Additionally, we examined the correlation between the risk model and the expression of HLA-associated genes, which have a vital function in presenting antigens and responding to the immune system. The findings indicated a notable association between the risk score and multiple HLA genes, suggesting the potential influence of the risk model on the presentation of antigens in ccRCC (**Figure 5J**).

### TCGA data mutation analysis

**Figure 6:**
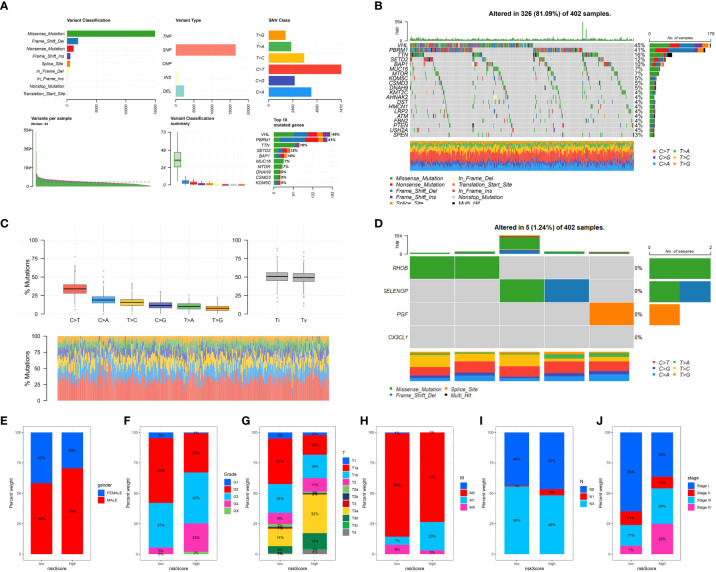
**(A)** Oncoplot illustrating the mutational landscape, where each column represents an individual patient sample and each row represents a gene. Color coding indicates the type of mutation (e.g., missense, frameshift, or truncation); **(B)** Overview of the most frequently mutated genes across the analyzed cancer types, with the percentage of samples affected shown in a descending order; **(C)** Mutation spectrum showing the distribution of the six possible base change categories across all mutations detected.; **(D)** The tumor mutation rate for specific genes in ccRCC cohort; The correlation between risk score and gender **(E)**, grade **(F)**, T stage **(G)**, M stage **(H)**, N stage **(I)** and stage **(J)**.

By analyzing the dataset from TCGA, we obtained valuable information about the mutational patterns in clear cell renal cell carcinoma (ccRCC). The distribution and classification of the most commonly mutated genes in ccRCC were demonstrated through a waterfall plot showcasing the mutation data. In ccRCC research, previous findings aligned with the top mutated genes including VHL, PBRM1, and TTN (**Figures 6A–D**).

### Risk model and clinical traits correlation

Afterwards, we investigated the correlation between the risk model and different clinical characteristics in patients with ccRCC. Several important clinical features, including tumor stage, grade, and gender, showed significant correlations with the risk score. Patients classified as high-risk displayed more advanced tumor stages and higher grades in comparison to those classified as low-risk. The findings indicate that our risk model has the potential to predict clinical outcomes in patients with ccRCC (**Figures 6E–J**).

### Analysis of GSVA using KEGG and HALLMARK concepts

**Figure 7:**
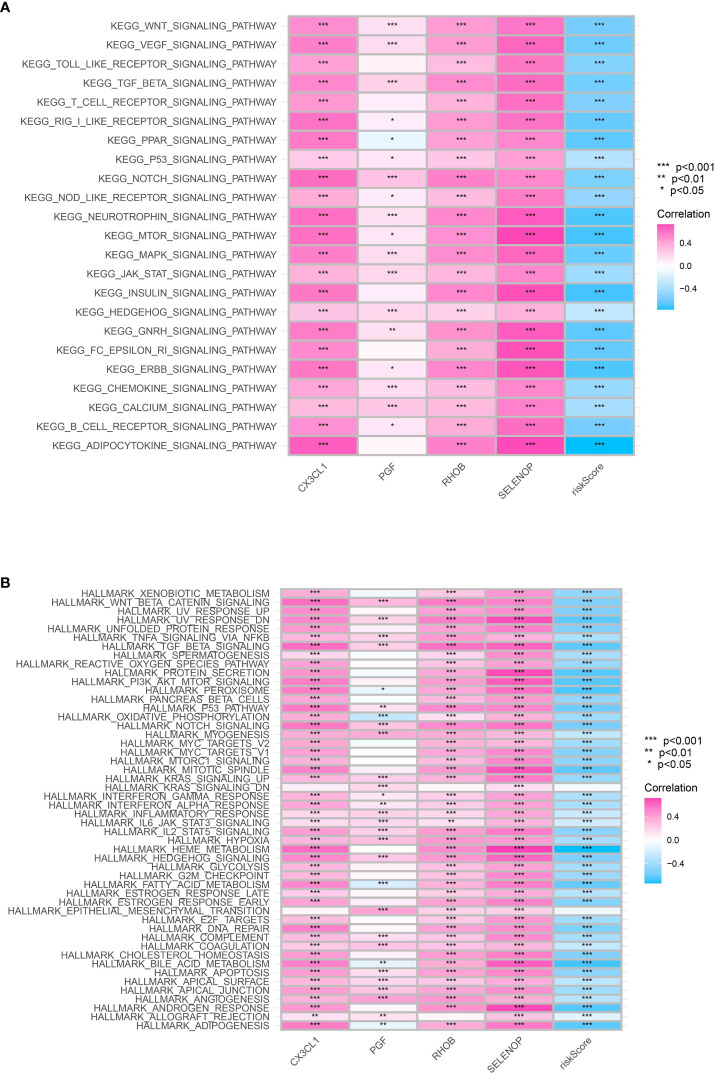
**(A)** The GSEA analysis based on the KEGG terms; **(B)** The GSEA analysis based on HALLMARK terms.

To enhance comprehension of the differentially expressed pathways within the KEGG (Kyoto Encyclopedia of Genes and Genomes) and HALLMARK gene sets, the utilization of Gene Set Variation Analysis (GSVA) was implemented. Our findings indicated a significant enrichment of genes associated with the JAK-STAT, MTOR, and MAPK signaling pathways in the high-risk category of KEGG pathways, implying an elevated rate of cell growth and genetic instability in this particular group (**Figure 7A**). In the HALLMARK gene sets, pathways associated with cancer such as the p53 pathway, E2F targets, and kras signaling pathway were discovered to be notably enriched in the low-risk group, which corresponds to the observed aggressive traits of this group (**Figure 7B**).

### Exploration of the role of CX3CL1 in renal cell carcinoma cells

**Figure 8:**
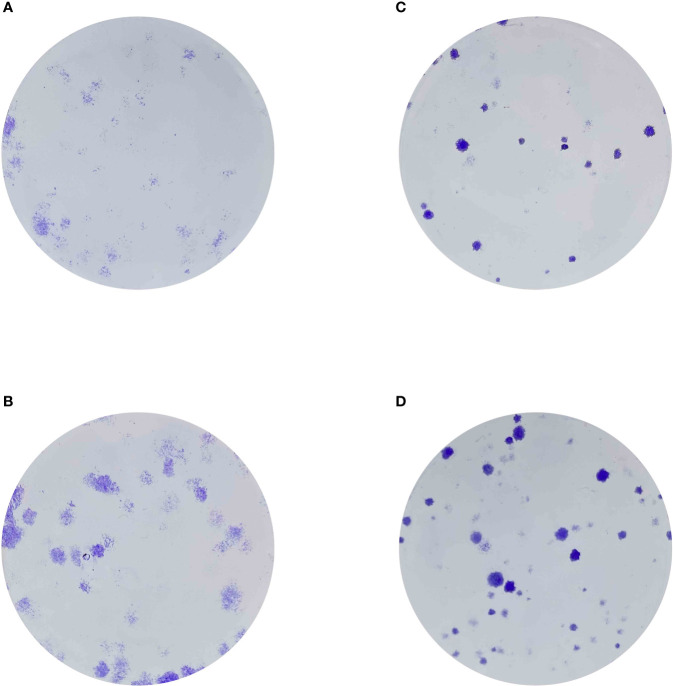
**(A)** The colony formation assay in 786-O cells; **(B)** The colony formation assay in 786-0 cells with the overexpression of CX3CL1; **(C)** The colony formation assay in Caki-1 cells; **(D)** The colony formation assay in Caki-1 cells with the overexpression of CX3CL1.

Finally, in order to evaluate the role of CX3CL1 in renal cell carcinoma cells, we then performed the cell proliferation assays. The results demonstrated that the over expression of CXCL1 could promote the cell proliferation ability in renal cell carcinoma cells (**Figure 8**).

## Discussion

The extensive examination has offered vital observations on the metabolic changes linked to clear cell renal cell carcinoma (ccRCC), specifically emphasizing the significance of genes related to diabetes. By employing ssGSEA, we have discovered a strong association between metabolic pathways and ccRCC. The levels of immune infiltration and the expression of genes related to HLA were associated with scores obtained from ssGSEA analysis of metabolism. Additionally, it is worth mentioning that our score related to metabolism exhibited promise in directing therapies associated with immune checkpoints, which could assist in formulating personalized and more efficient treatment approaches for patients with ccRCC.

Furthermore, we utilized differential expression analysis to detect genes associated with diabetes and metabolism in ccRCC, thereby enhancing our comprehension of the metabolic irregularities in ccRCC. The ESTIMATE algorithm offered a thorough comprehension of the immune scenery in the ccRCC microenvironment, providing intriguing indications of potential immune evasion mechanisms in patients at high risk.

By utilizing the Weighted Gene Co-expression Network Analysis (WGCNA), we enhanced our comprehension of the immune environment of ccRCC. This analysis revealed the involvement of various immune-related cells in regulating the immune response. Crucially, the gene module obtained from WGCNA exhibited a correlation with the survival of patients, underscoring the clinical significance of the identified immune-related genes.

Our constructed prognostic model yielded significant prognostic markers for ccRCC, and the associated risk scores seemed to function as a standalone prognostic determinant. The reliability and precision of this model were additionally confirmed by conducting ROC curve analysis and developing a prognostic nomogram.

The clinical significance of our findings was enhanced by our examination of TCGA mutation data and the association between the risk model and different clinical characteristics. Collectively, our research offers a significant basis for comprehending the complex connection between metabolic alterations associated with diabetes and ccRCC. It underscores the necessity for future studies to further investigate these metabolic pathways, with the aim of developing novel therapeutic strategies that target these metabolic abnormalities in ccRCC patients.

Nevertheless, there are constraints to take into account in this research. The examinations were performed on information obtained from a solitary group, thus, forthcoming investigations should strive to authenticate these discoveries in separate groups. Furthermore, it is necessary to conduct mechanistic investigations in order to elucidate the precise mechanisms that underlie the observed correlations. In spite of these constraints, our research presents a valuable viewpoint on the metabolic imbalance in ccRCC and offers potential indicators for prognosis and targets for therapeutic investigation.

## Data availability statement

The original contributions presented in the study are included in the article/supplementary material. Further inquiries can be directed to the corresponding author.

## Author contributions

XC: Conceptualization, Investigation, Writing – original draft. YH: Methodology, Supervision, Writing – review & editing.
