# Supplementary figures and images for: Importance of metabolic and immune profile as a prognostic indicator in patients with diabetic clear cell renal cell carcinoma
Source: Front Oncol. 2023 Oct 20;13:1280618. doi: 10.3389/fonc.2023.1280618 (PMC10623455; doi:10.3389/fonc.2023.1280618)

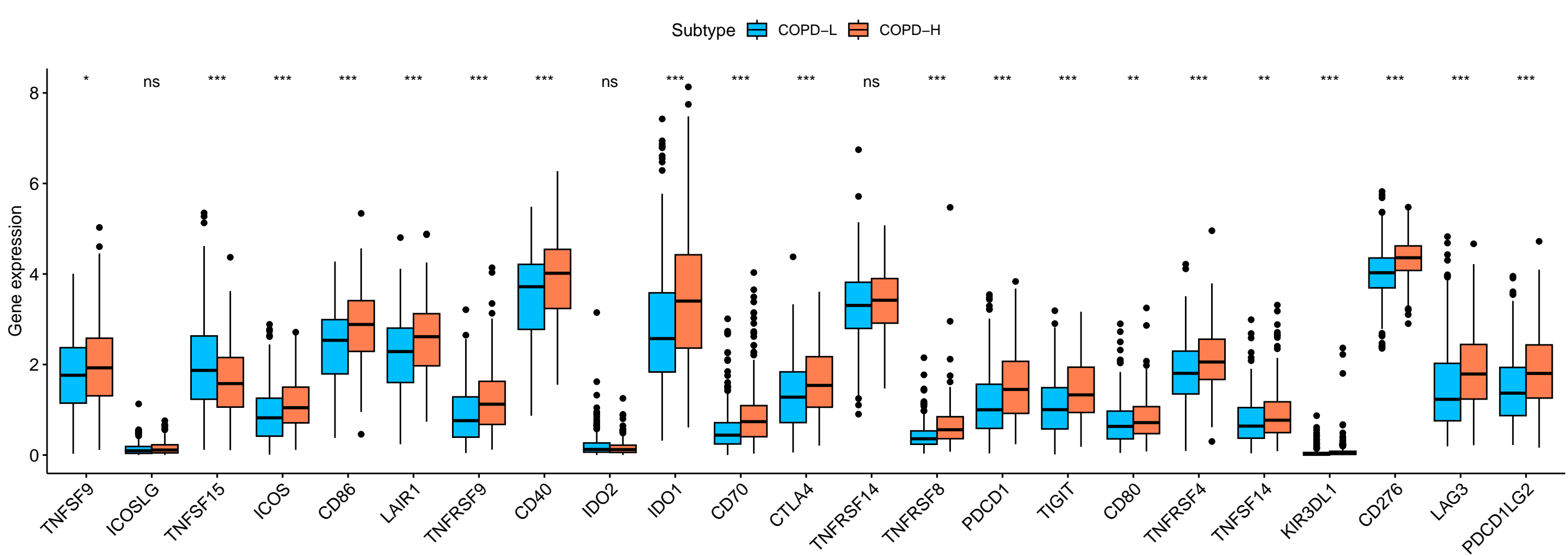

Supplement: Supplementary file 3 [file DataSheet_3.zip › figure 3/ckp.pdf]

Subtype COPD-L COPD-H

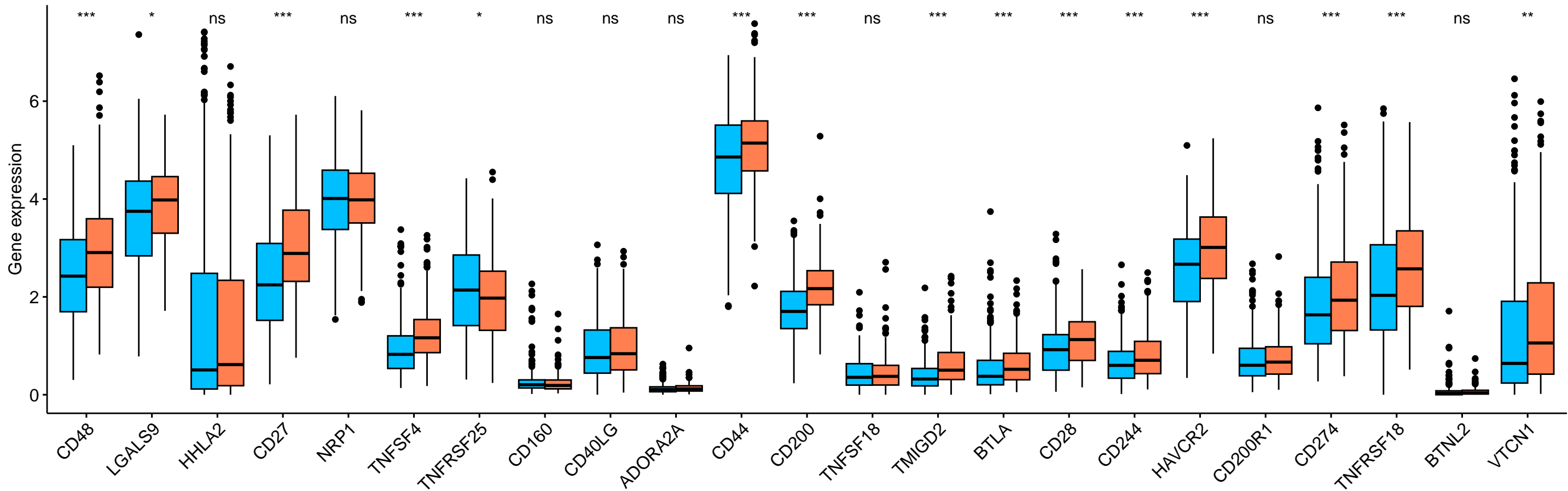

Supplement: Supplementary file 3 [file DataSheet_3.zip › figure 3/ckp2.pdf]

Subtype COPD-L COPD-H

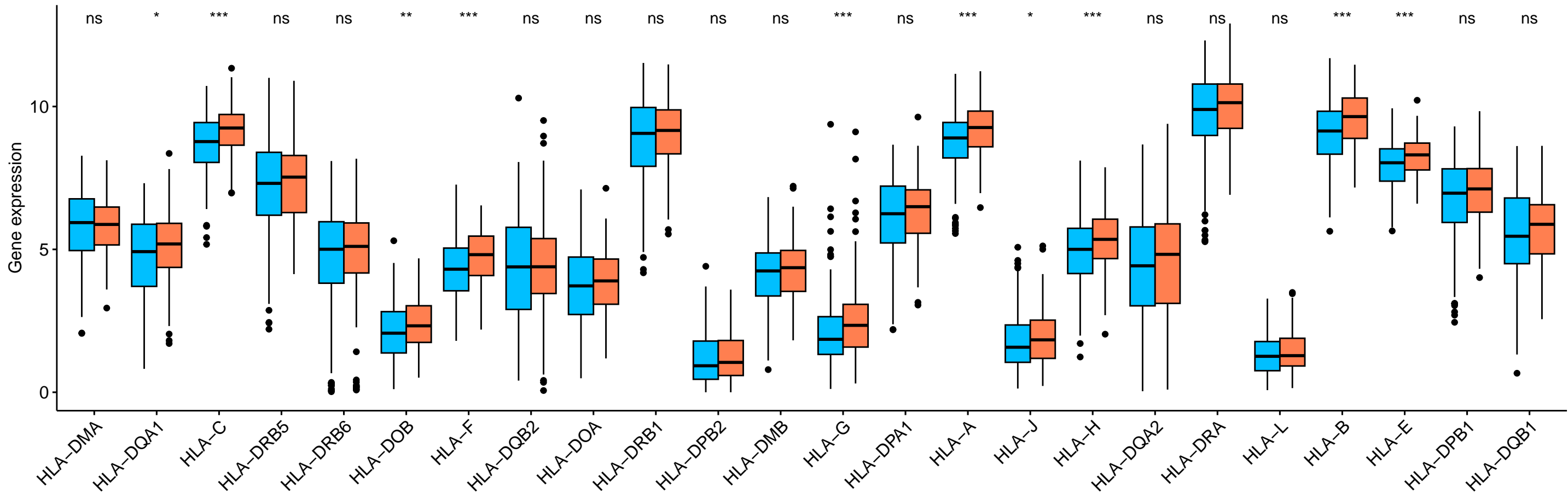

Supplement: Supplementary file 3 [file DataSheet_3.zip › figure 3/HLA.pdf]

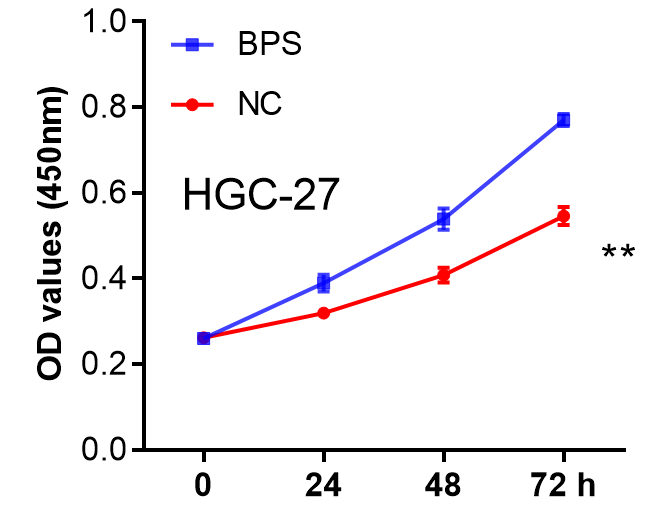

Supplement: Supplementary file 8 [file DataSheet_8.zip › figure 9/CCK8/39e33f31fc0614e4463f7e7a2b44674.png]

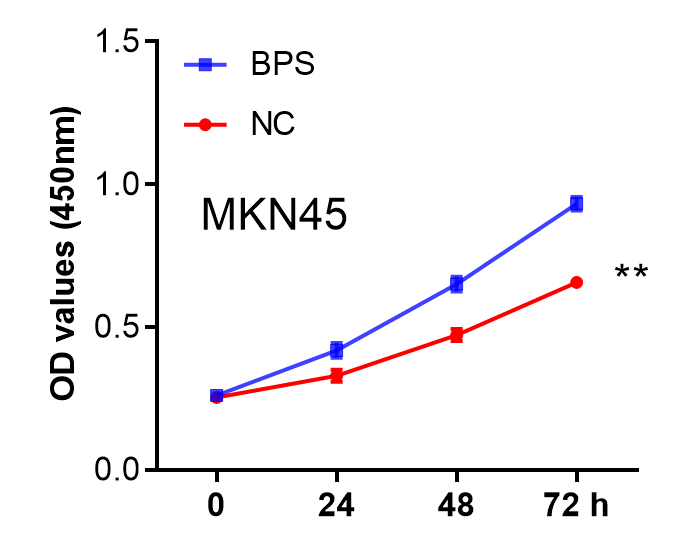

Supplement: Supplementary file 8 [file DataSheet_8.zip › figure 9/CCK8/8897f8a597d3a71d8b8776cb68221db.png]

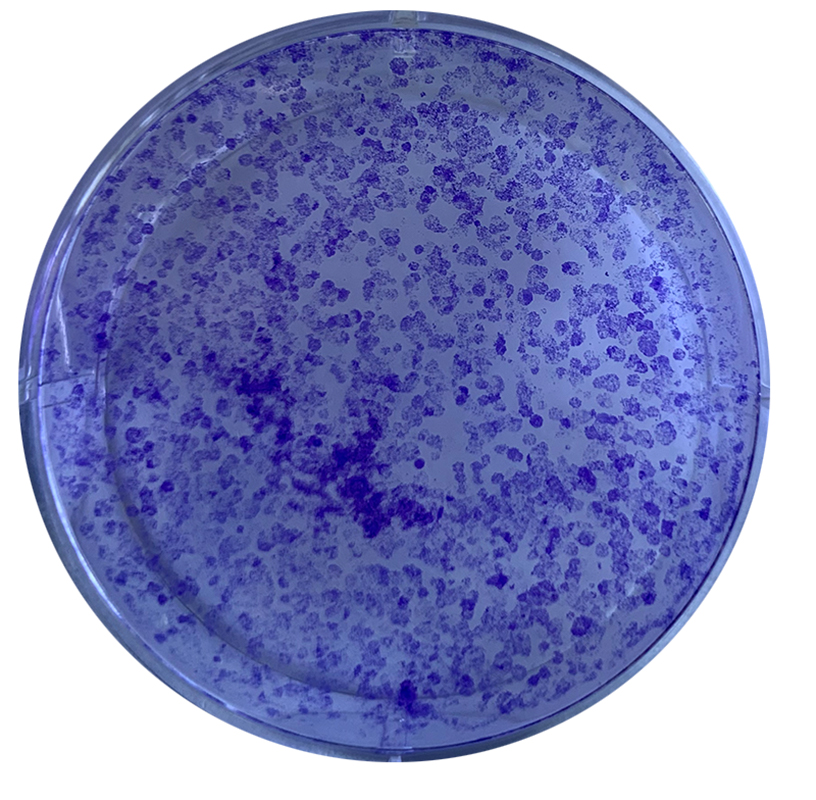

Supplement: Supplementary file 8 [file DataSheet_8.zip › figure 9/COLONY/HGC 27 NC.jpg]

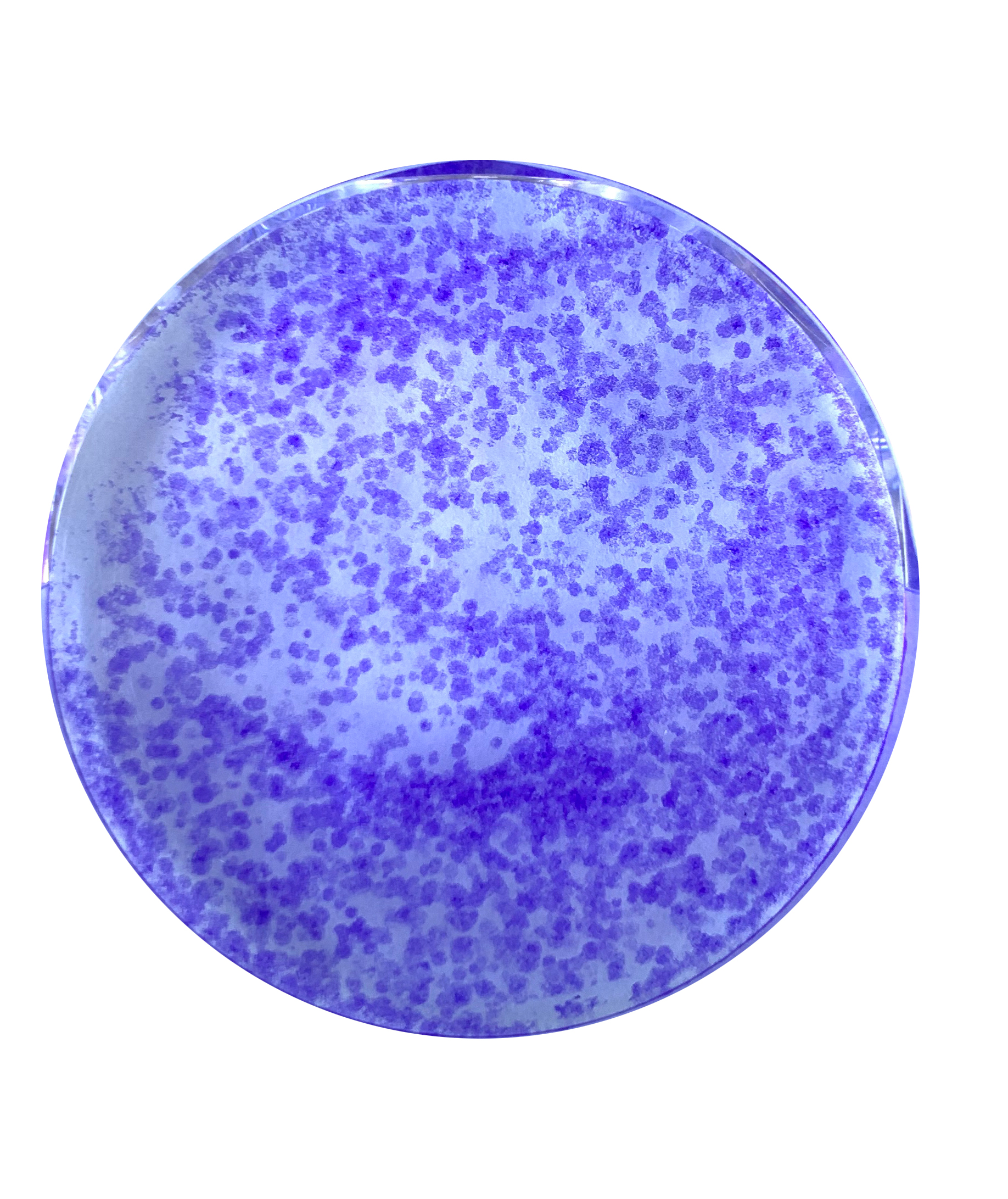

Supplement: Supplementary file 8 [file DataSheet_8.zip › figure 9/COLONY/hgc27 bps.jpg]

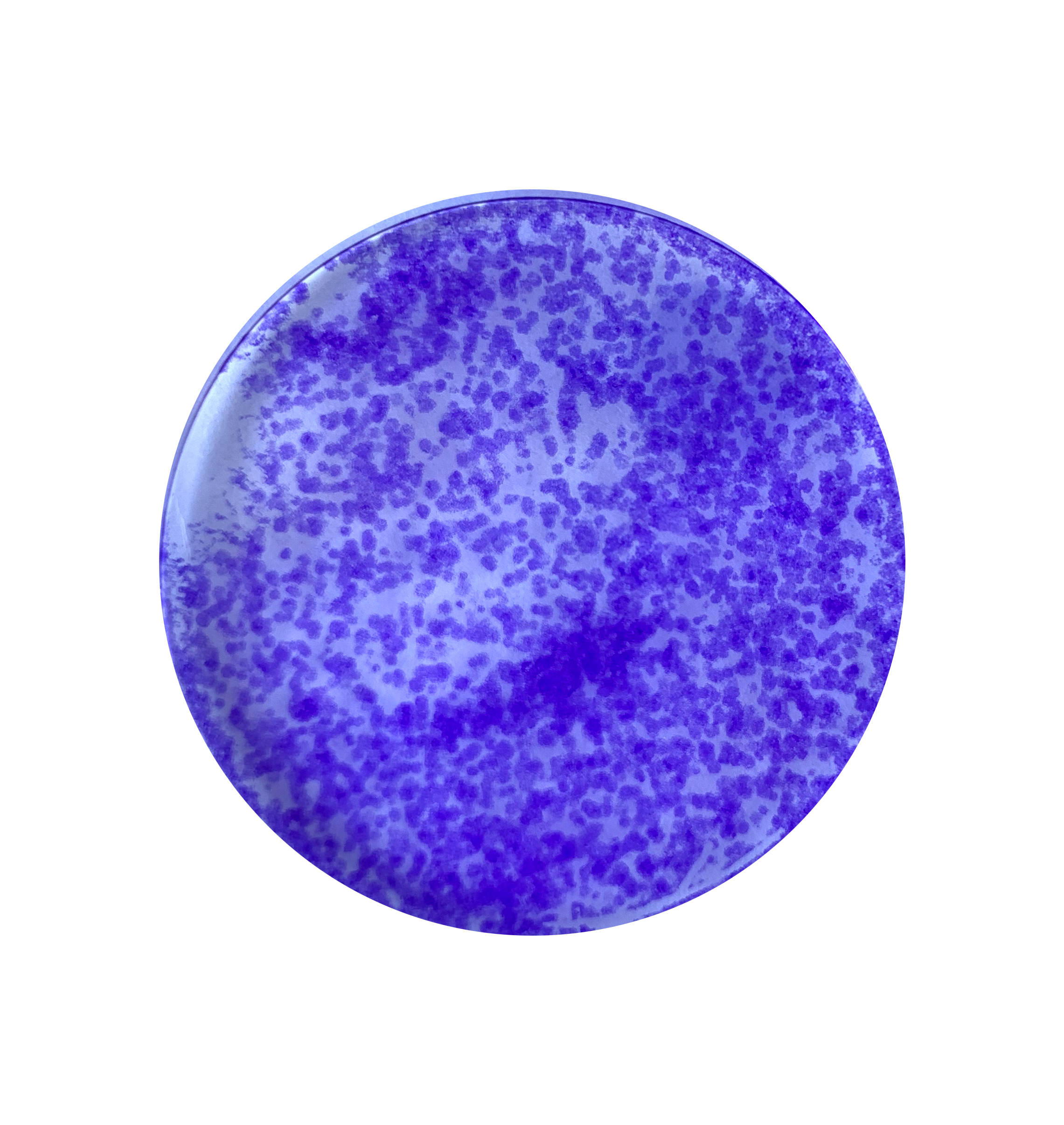

Supplement: Supplementary file 8 [file DataSheet_8.zip › figure 9/COLONY/mkn45 bps.jpg]

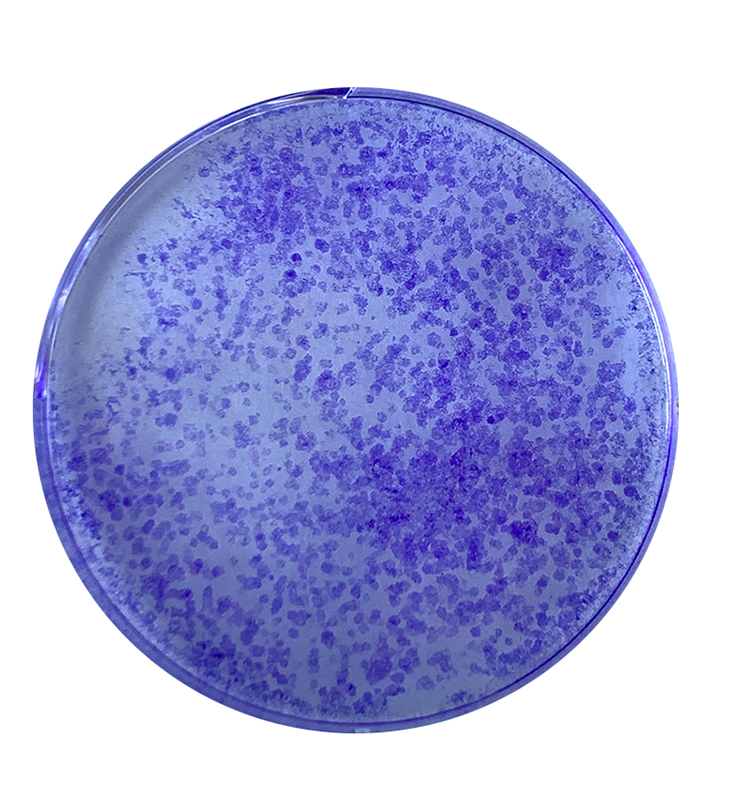

Supplement: Supplementary file 8 [file DataSheet_8.zip › figure 9/COLONY/MKN45 NC.jpg]

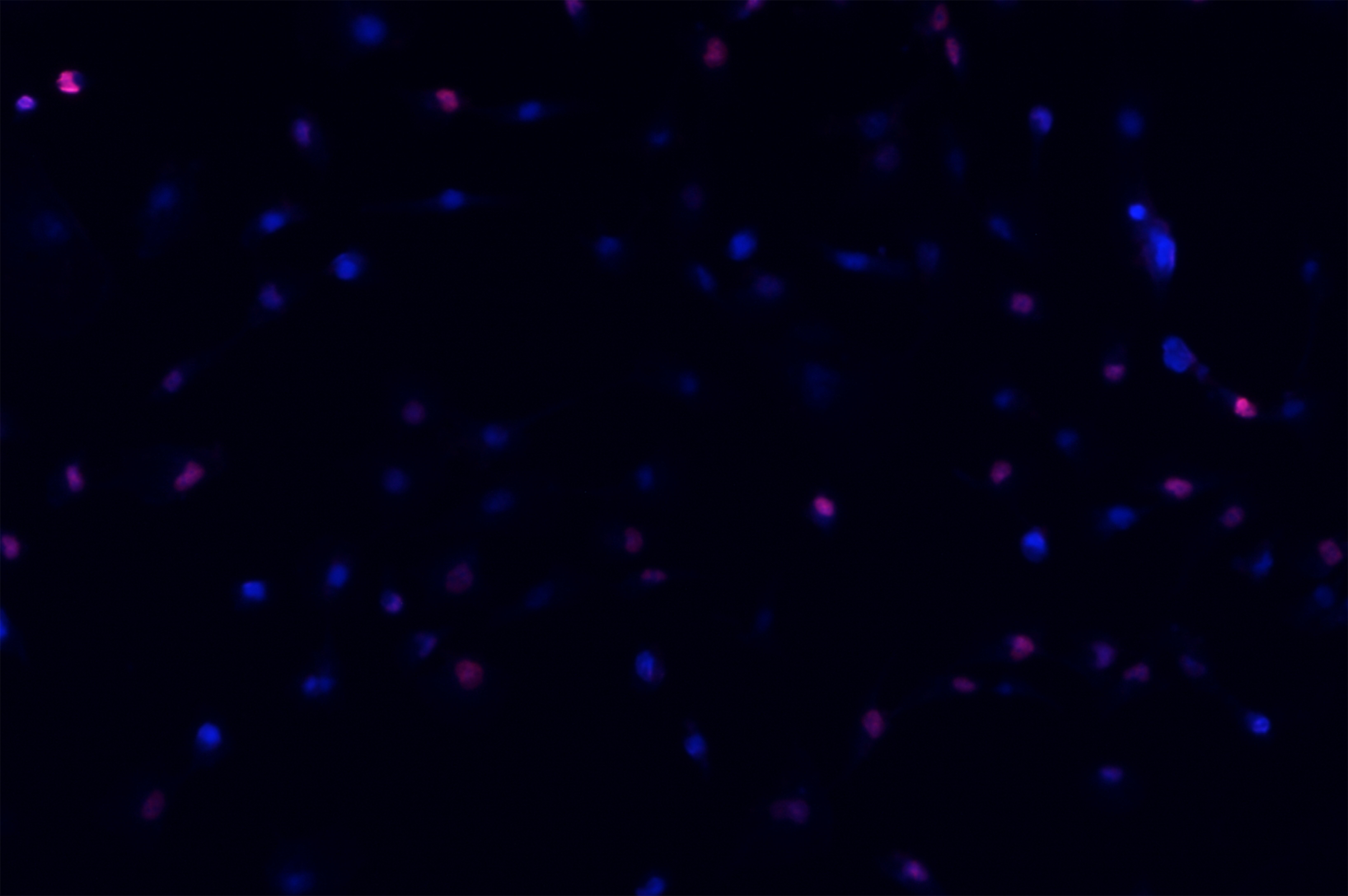

Supplement: Supplementary file 8 [file DataSheet_8.zip › figure 9/Edu/NC HGC MERGE.tif]

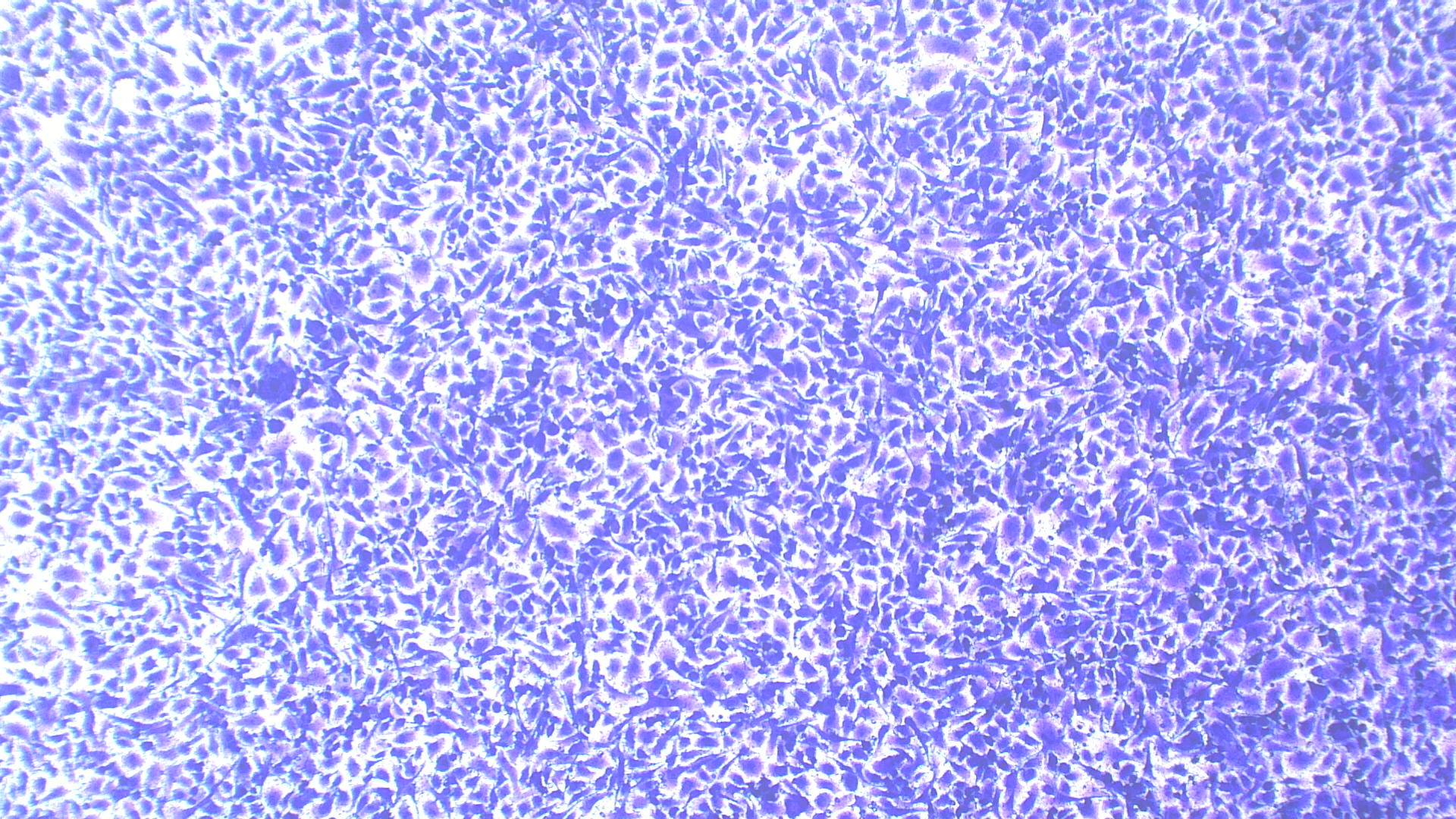

Supplement: Supplementary file 8 [file DataSheet_8.zip › figure 9/transwell/HGC27-BPS.tif]

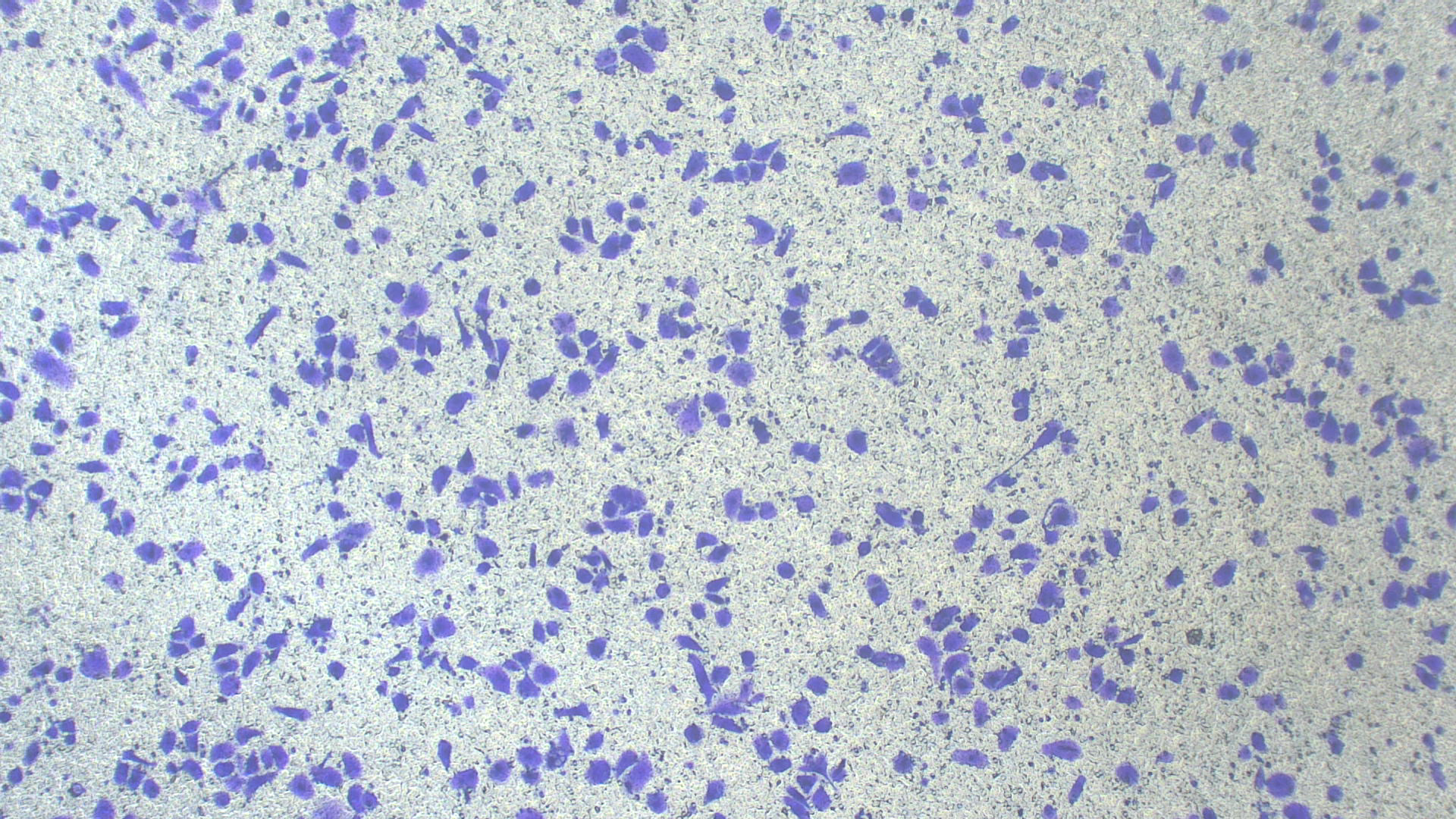

Supplement: Supplementary file 8 [file DataSheet_8.zip › figure 9/transwell/HGC27-NC.tif]
